# Supplementary material for: Tailored Uniaxial Alignment of Nanowires Based on Off-Center Spin-Coating for Flexible and Transparent Field-Effect Transistors
Source: Nanomaterials (Basel). 2022 Mar 28;12(7):1116. doi: 10.3390/nano12071116 (PMC9000857; doi:10.3390/nano12071116)
Supplement: Supplementary file 1 [file nanomaterials-12-01116-s001.zip › nanomaterials-1639301-supplementary.pdf]

Supporting Information

Tailored Uniaxial Alignment of Nanowires Based on Off-Center Spin-Coating for Flexible and Transparent Field-Effect Transistors

Giwon Lee [1,†], Haena Kim [1,‡], Seon Baek Lee [1,†], Daegun Kim [1], Eunho Lee [2], Seong Kyu Lee [1] and Seung Goo Lee [3,*]

[1] Department of Chemical Engineering, Pohang University of Science and Technology, Pohang 37673, Korea; giwonlee@postech.ac.kr (G.L.); haenak@postech.ac.kr (H.K.); sbree@postech.ac.kr (S.B.L.); daegunkim@postech.ac.kr (D.K.); sklee84@postech.ac.kr (S.K.L.)
[2] Department of Chemical Engineering, Kumoh National Institute of Technology, Gumi 39177, Korea; leeeh@kumoh.ac.kr
[3] Department of Chemistry, University of Ulsan, Ulsan 44610, Korea
* Correspondence: lees9@ulsan.ac.kr

† These authors contributed equally to this work.

1. Methods

1.1. Synthesis of the Si NWs

The vertically-aligned Si NWs were initially synthesized via a metal-assisted chemical etching technique (**Figure S1**). For this procedure, commercial Si wafers (p-type, <100> oriented, 0.5–0.75Ωcm) were cleaned using piranha solution (1:3 wt/wt H_2O_2:H_2SO_4, 120 °C) for 20 min, followed by etching for 5 min with 10% HF aqueous solution to remove the oxide layer and generate H-terminated surfaces. The fresh Si wafers were then removed from this solution and immediately immersed in a solution containing 20% HF and 0.04 M $AgNO_3$ for 50 s for surface deposition of Ag particles The wafers were then washed with deionized water to remove any extra Ag^+ ions, and immersed in an etchant containing 20% HF and 1.2 wt.% H_2O_2 at 50 °C. After etching in the dark for 150 min, the Si wafers were dipped in a 10% HF aqueous solution to remove the resulting oxide layer, then washed with DI water, and dried under a flow of N_2 gas.

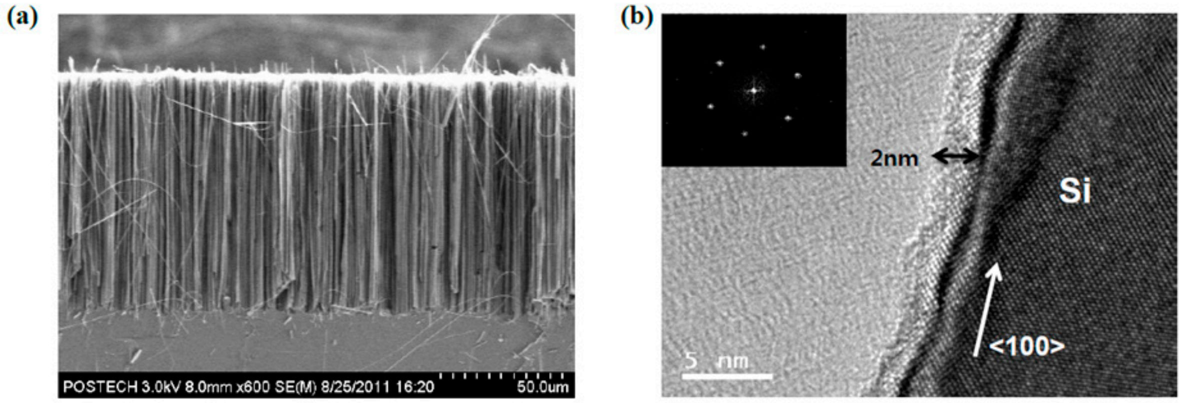

Figure S1. Characterization of the Si NWs prepared by metal-assisted chemical etching: (a) a cross-sectional SEM image of the ~100-μm Si NWs obtained by 150 min of etching; (b) a high-resolution TEM image and selected area electron diffraction pattern (inset) of an Si nanowire.

1.2. Fabrication and characterization of the Si NW FET

The NWs produced via metal-assisted chemical etching were cut and dispersed in isopropyl alcohol (IPA) via sonication for 10 min. The dispersion was then left to stand on a flat table for one day, thus allowing the heavy Si NW groups and bundles to sink to the bottom, while the light individual Si NWs remained dispersed in the IPA. This upper dispersion was separated from the sunken Si NWs and centrifuged at 7000 rpm for 20 min to remove any residual ions such as Ag^+, H^+, F^-. The obtained NWs were subsequently dispersed in various alcohol solvents and used for the spin-coating process.

The various dispersions were coated onto n-doped Si substrates with 300 nm thick SiO_2 gate dielectrics via the new spin-coating process. The source and drain electrodes were then deposited by thermal evaporation of Au (100 nm). For the fabrication of flexible Si NW FETs, a 2-μm parylene-C layer was thermally deposited onto an Si wafer as the sacrificial substrate (OBTPC 500, Obang Technology).[2] A polyimide (PI) layer was then coated onto the substrate as the planarization layer. The Al gate electrodes (40 nm) were then thermally evaporated, the parylene-C was vacuum-deposited as the gate dielectric layer, the Si NWs were coated via the above-mentioned method, and the Au source/drain electrodes were thermally evaporated through a shadow mask. The current-voltage characteristics of the Si NW FETs were measured at room temperature under ambient conditions.

1.3. Material Characterization

The morphologies of samples were characterized using field emission scanning electron microscopy (FESEM) (Hitachi S-4200), transmission electron microscopy (TEM) (JEM-2200FS), and optical microscopy (OM) (Axioplan Zeiss). The contact angle was analyzed using a contact-angle meter (SmartDrop Plus, Femtobiomed). The electrical properties of the field-effect transistor, current-voltage (*I–V*) curves, and current bias were measured using a Keithley 2400 and S4200 instrument. All procedures were approved by the Research Ethics Committee of Pohang University of Science and Technology in South Korea (PIRB-2020-E017). Written informed consents were obtained from all subjects.

2. Modification of the formulas for the drag and centrifugal forces

2.1. Drag force

The Reynolds number (Re) of the Si nanowire system is given by:

$$\mathrm{Re} = \frac{\rho l(v-u)}{\mu} \approx 10^2$$

Laminar flow

In the case of laminar flow, the relationship between Re and the coefficient of frictional drag (C_{Df}) is given by:

$$C_{Df} = \frac{1.328}{\sqrt{Re}}$$

and the friction drag force is given by:

$$F_{Df} = \frac{1}{2}\rho A C_{Df}(v-u)^2$$

$$= \frac{1}{2}\rho\pi R l C_{Df}(v-u)^2$$

$$= \frac{1}{2}Re\mu\pi R C_{Df}(v-u)$$

$$= \frac{1}{2}\pi R\mu\frac{1.328^2}{C_{Df}}(v-u) \approx \frac{\pi R\mu}{C_{Df}}(v-u) \quad \mu \propto F_{Df}\,, \ (v-u) \propto F_{Df}$$

where R is the radius of the NW, μ is the viscosity of the solvent, v is the velocity of the nanowire, and u is the fluid velocity.

2.2. Centrifugal force

According to Marchell *et al.*[1], the centrifugal force (F_c) is given by:

$$F_c = (\chi - 1)m_N{\Omega_s}^2 r; \qquad \chi = \frac{\rho_f}{\rho_p}$$

where m_N is the mass of the NW, Ω_S is the angular rotation of the substrate, and r is the distance between the substrate and the axis of roation.

In the present system, ρ_P can can be replaced by ρ_N (the density of an Si NW) and, as the density of the solvent is very similar, χ can be omitted. This gives the modified centrifugal force equation:

$$F_c = m_N{\Omega_s}^2 r$$

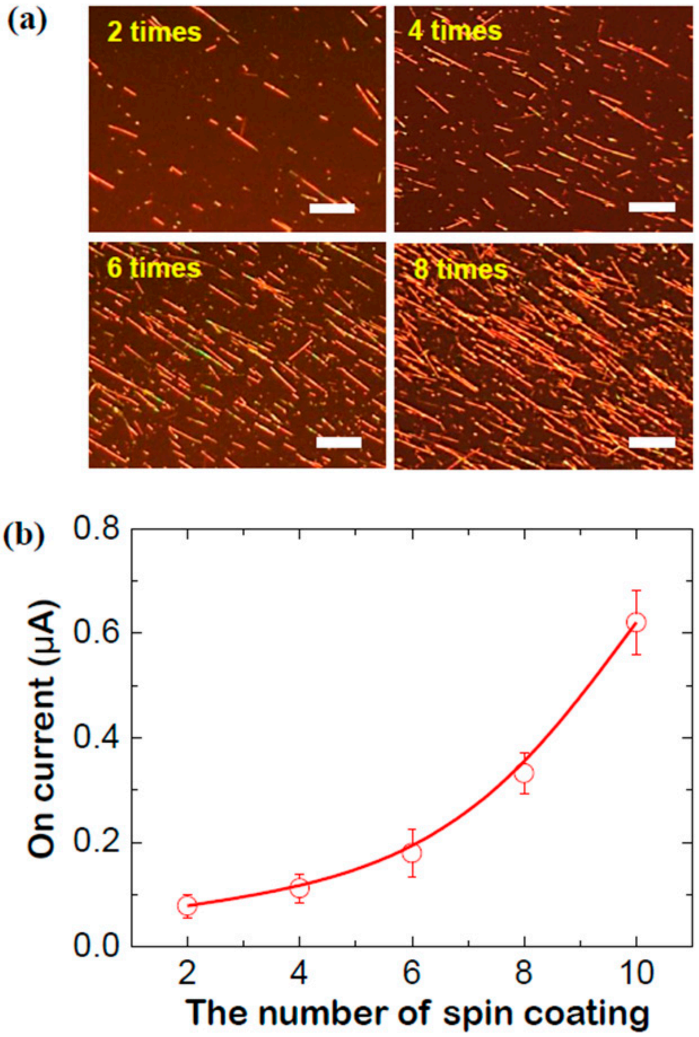

Figure S2 (a) The dark-field optical images of the aligned Si NWs dispersed in isobutanol (0.1 wt.%) after 2, 4, 6, and 8 repeat spin-coating processes (scale bar = 50 μm). (b) A plot of the on current against number of repeat spin-coating processes.

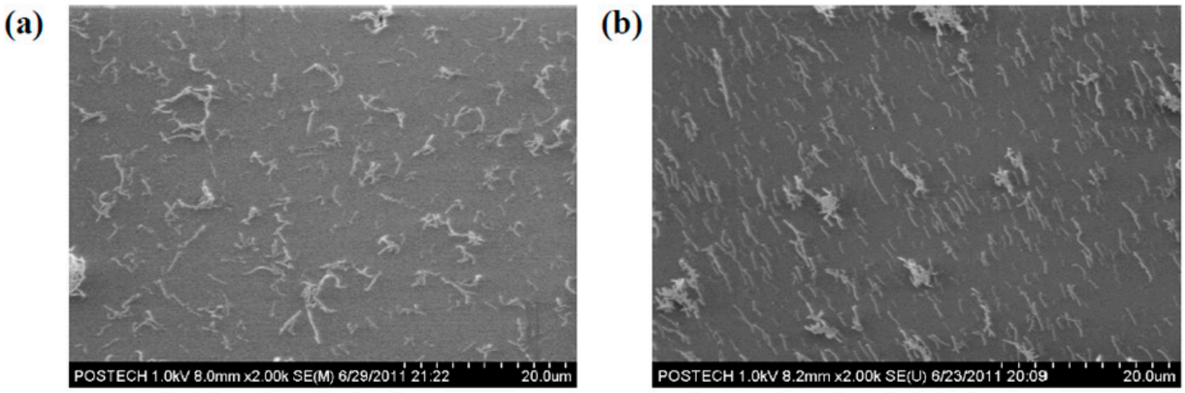

Figure S3 The SEM images of single-walled carbon nanotubes (SWCNTs) dispersed in (a) methanol, and (b) 1:1 vol/vol methanol and n-butanol after the spin-assisted alignment process.

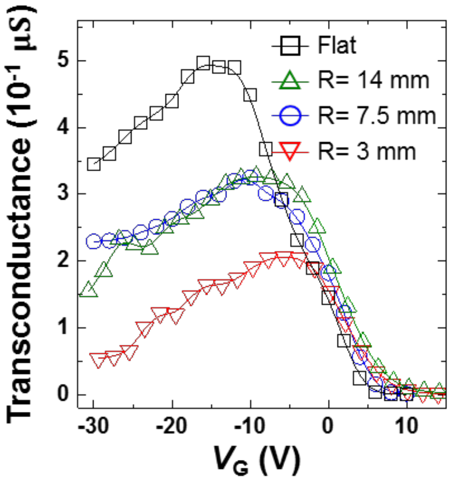

Figure S4 Plots of the transconductance against gate voltage (V_G) for various bending radii of the flexible device.

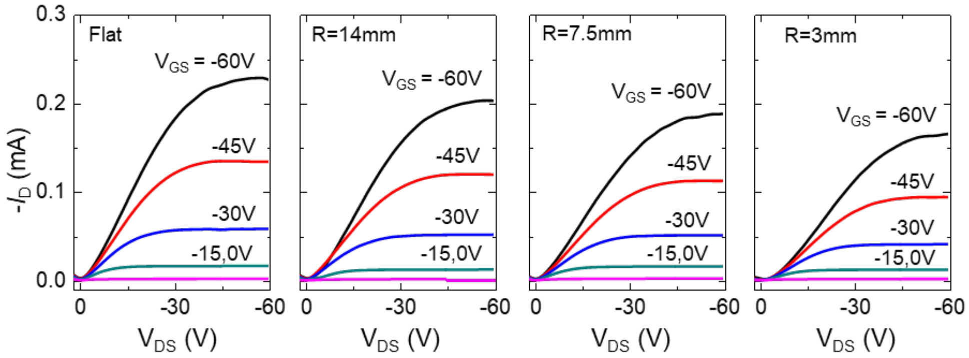

Figure S5 The electrical characteristics of output curve of the flexible devices attached to the various surfaces.

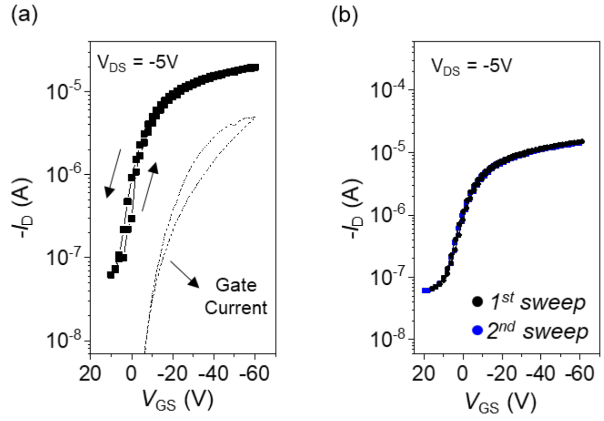

Figure S6 The electrical characteristic of transfer curve of the flexible device with (a) dual sweep (V_{DS} = -5V) and (b) twice sweep.

References

1. Zhao, Y.; Marshall, J. S. Spin Coating of a Colloidal Suspension. *Phys. Fluids* **2008**, 20, 043302.
2. Lee, S. B.; Kang, B.; Kim, D.; Park, C.; Kim, S.; Lee, M.; Lee, W. B.; Cho, K. Motion-Programmed Bar-Coating Method with Controlled Gap for High-Speed Scalable Preparation of Highly Crystalline Organic Semiconductor Thin Films. *ACS Appl. Mater. Interfaces* **2019**, 11, 47153-47161.
3. Liu Z.; Yin Z.; Wang J.; Zheng Q. Polyelectrolyte Dielectrics for Flexible Low-Voltage Organic Thin-Film Transistors in Highly Sensitive Pressure Sensing. *Adv. Funct. Mater.* **2019**, 29, 1806092.
